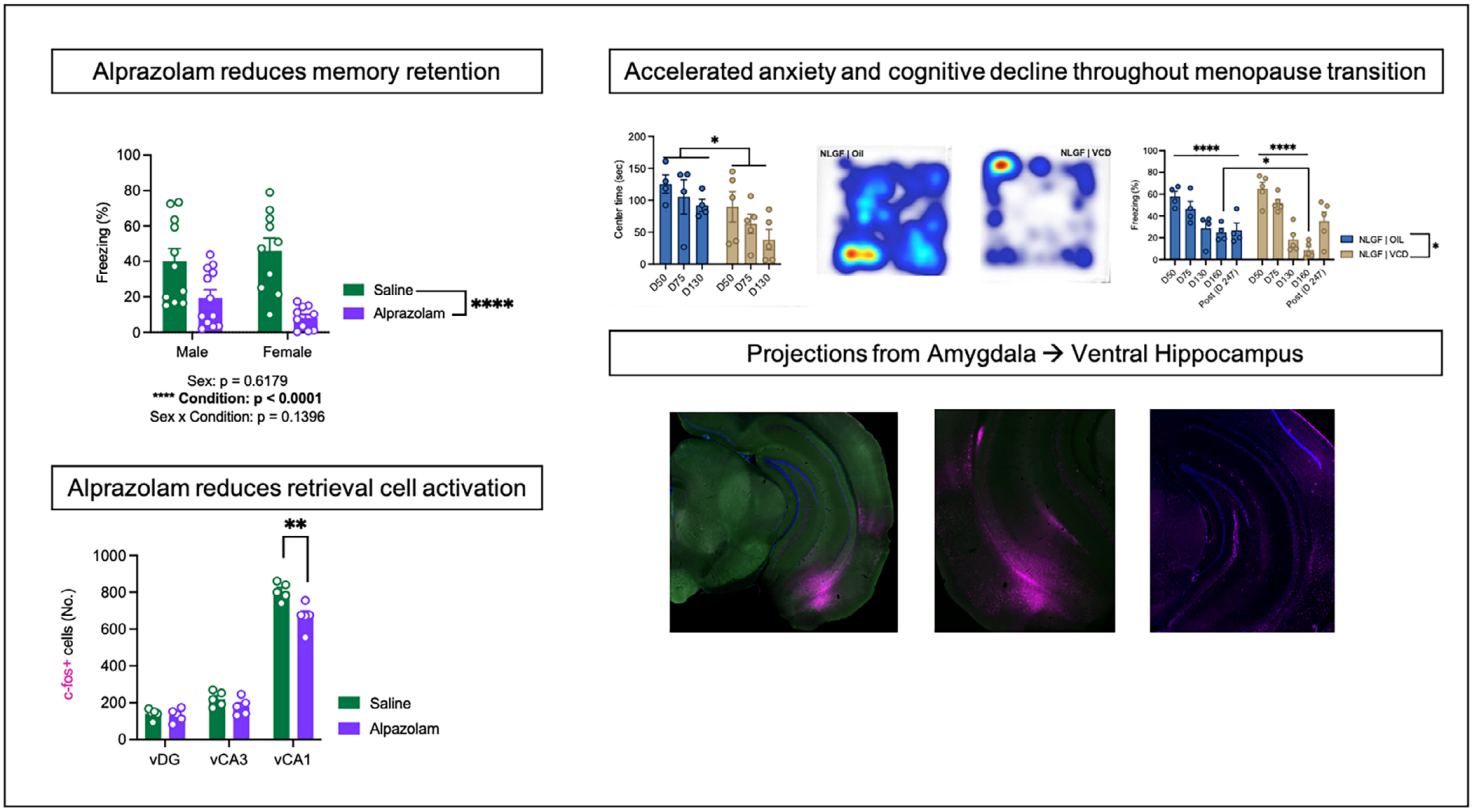# Understanding and targeting neuropsychiatric symptoms in aging, Alzheimer's, and throughout menopause

**DOI:** 10.1002/alz70855_101072

**Published:** 2025-12-23

**Authors:** Holly C Hunsberger, Kameron Kaplan, Lainey B Toennies

**Affiliations:** ^1^ Rosalind Franklin University, North Chicago, IL, USA; ^2^ The Chicago Medical School, North Chicago, IL, USA

## Abstract

**Background:**

Our preliminary work revealed earlier anxiety and cognitive decline in female Alzheimer's disease (APP/PS1) mice which correlated to an unbalanced brain‐wide network. To validate these preclinical findings, we analyzed the impact of anxiety on AD in human subjects using the Alzheimer's disease neuroimaging (ADNI) dataset. Our analysis showed that that 1) female AD subjects exhibited higher anxiety; 2) female subjects with amyloid deposition transition to dementia at a faster rate compared to male subjects; 3) female subjects with anxiety have smaller brain volumes; and 4) anxiety is the best predictor of dementia transition. Here, we examine the impact of treating mice with anxiolytics, dissect the circuitry controlling anxiety, and determine whether menopause accelerates AD pathology.

**Method:**

Alprazolam (Xanax) was injected 30 minutes prior to a contextual fear memory task in control male and female mice. We then took brain tissue 60 minutes after testing to measure neuronal ensembles activated within the hippocampus. In control and AD (APP/PS1) mice, Cholera Toxin B, was injected into the amygdala and lateral hypothalamus. We measured projections to the ventral hippocampus at 2 and 6 months of age. Lastly, we induced menopause using a chemical, VCD, which depletes ovarian follicles similar to the human transitions. We then ran these mice through a battery of anxiogenic behavior paradigms and examined memory.

**Result:**

We found that Alpazolam‐treated male and female mice exhibit a decrease in memory retention, most likely the result of less retrieval cell activation in ventral hippocampus. In mice transitioning through perimenopause we observed earlier anxiety‐like behavior and cognitive decline, but this decline rebounded in postmenopause suggesting a compensatory mechanism. We predict that there will be an increase in the number of projections in ventral hippocampus in female AD mice compared to controls and males.

**Conclusion:**

These findings suggest that Neuropsychiatric symptoms impact cognitive decline through sex‐specific pathways in the hippocampus and that benzodiazepines may not be beneficial when treating Alzheimer's disease patients. Menopause can also induce this earlier anxiety‐like phenotype, but the brain may compensate for these hormonal changes. Future studies will examine the impact of chronic BZD use on aging and Alzheimer's disease.